# Atomic Structure of the Trichomonas vaginalis Double-Stranded RNA Virus 2

**DOI:** 10.1128/mBio.02924-20

**Published:** 2021-03-30

**Authors:** Alexander Stevens, Katherine Muratore, Yanxiang Cui, Patricia J. Johnson, Z. Hong Zhou

**Affiliations:** a Department of Microbiology, Immunology & Molecular Genetics, University of California, Los Angeles, Los Angeles, California, USA; b California NanoSystems Institute, University of California, Los Angeles, Los Angeles, California, USA; c Department of Chemistry and Biochemistry, University of California, Los Angeles, Los Angeles, California, USA; Columbia University

**Keywords:** cryoEM, subparticle reconstruction, double-stranded RNA virus, *Trichomonas vaginalis*, *Totiviridae*

## Abstract

Trichomonas vaginalis, the causative pathogen for the most common nonviral sexually transmitted infection worldwide, is itself frequently infected with one or more of the four types of small double-stranded RNA (dsRNA) Trichomonas vaginalis viruses (TVV1 to 4, genus *Trichomonasvirus*, family *Totiviridae*). Each TVV encloses a nonsegmented genome within a single-layered capsid and replicates entirely intracellularly, like many dsRNA viruses, and unlike those in the *Reoviridae* family. Here, we have determined the structure of TVV2 by cryo-electron microscopy (cryoEM) at 3.6 Å resolution and derived an atomic model of its capsid. TVV2 has an icosahedral, T = 2*, capsid comprised of 60 copies of the icosahedral asymmetric unit (a dimer of the two capsid shell protein [CSP] conformers, CSP-A and CSP-B), typical of icosahedral dsRNA virus capsids. However, unlike the robust CSP-interlocking interactions such as the use of auxiliary “clamping” proteins among *Reoviridae*, only lateral CSP interactions are observed in TVV2, consistent with an assembly strategy optimized for TVVs’ intracellular-only replication cycles within their protozoan host. The atomic model reveals both a mostly negatively charged capsid interior, which is conducive to movement of the loosely packed genome, and channels at the 5-fold vertices, which we suggest as routes of mRNA release during transcription. Structural comparison of TVV2 to the Saccharomyces cerevisiae L-A virus reveals a conserved helix-rich fold within the CSP and putative guanylyltransferase domain along the capsid exterior, suggesting conserved mRNA maintenance strategies among *Totiviridae*. This first atomic structure of a TVV provides a framework to guide future biochemical investigations into the interplay between Trichomonas vaginalis and its viruses.

## Abstract (precis)

Trichomonas vaginalis viruses (TVVs) are double-stranded RNA (dsRNA) viruses that cohabitate in Trichomonas vaginalis, the causative pathogen of trichomoniasis, the most common nonviral sexually transmitted disease worldwide. Featuring an unsegmented dsRNA genome encoding a single capsid shell protein (CSP), TVVs contrast with multisegmented dsRNA viruses, such as the diarrhea-causing rotavirus, whose larger genome is split into 10 dsRNA segments encoding 5 unique capsid proteins.

## INTRODUCTION

Trichomonas vaginalis (Tv) is a flagellated, parasitic protozoan responsible for the most common nonviral sexually transmitted infection (STI) worldwide, trichomoniasis, which afflicts nearly 280 million people each year (1, 2). Tv infection is associated with increased rates of developing aggressive genitourinary cancers, adverse pregnancy outcomes, and transmitting other STIs, including human immunodeficiency virus (3–11). Clinical Tv isolates are frequently found infected with one or more strain of the double-stranded RNA (dsRNA) Trichomonas vaginalis viruses (TVVs), belonging to the *Trichomonasvirus* genus within totiviruses (12, 13). The four phylogenetically distinct TVV strains (TVV1 to 4) share little sequence identity (<28%) and do not transmit extracellularly but are found to cohabitate the same organism (12–18). TVV infection has been reportedly linked to upregulation of cysteine proteases and the major surface antigen, P270 in Tv, thought to contribute to subversion of the host immune system (19, 20). However, whether these effects are due to the virus or the protozoan strain remains unclear. This poorly understood relationship between TVVs and their pathogenic host has been compounded by the lack of detailed structural information which could help to interpret both clinical and laboratory observations.

TVV1 to 4 enclose 4.6 to 4.9 kbp unsegmented genomes in single-layered icosahedral capsid shells (capsids) (12). These TVV genomes contain two overlapping open reading frames encoding the 678 to 746 amino acid (aa) capsid shell protein (CSP) (74 to 82 kDa) and RNA-dependent RNA polymerase (RdRp) (12, 14–16). Due to ribosomal frameshifting, the RdRp complex is expressed as a C-terminal fusion protein (CSP-pol) of 1,429 to 1,481 aa’s (combined, 159 to 165 kDa) (12, 14–16). Similarly, many other cytosol-bound dsRNA viruses exhibit this simplistic morphology, including members of the *Chrysoviridae*, *Partitiviridae*, and *Quadriviridae* families (21–23). Because dsRNA is a powerful inducer of eukaryotic antiviral defenses (24), the capsids of dsRNA viruses often continuously conceal the viral genome to prevent detection by the host. In Tv, the burden of antiviral activity may fall on the putative RNA interference (RNAi) pathway of the protist, requiring passive dsRNA detection and providing limited downstream antiviral consequence (24–26). As the dsRNA genomes remain inside the capsid, internal RdRp complexes are necessary to synthesize positive-stranded viral mRNA to be exported to the cytosol via capsid spanning channels of various complexities. An earlier cryo-electron microscopy (cryoEM) study of the TVV1 virion resolved the icosahedral viral capsid to 6.7 to 5.5 Å resolution and observed an icosahedral, T = 2*, capsid composed of 60 asymmetric dimers and measuring 450 to 375 Å in diameter (27). The authors identified pores spanning the entire capsid at the 5-fold icosahedral (I5) vertices that were of reasonable diameter to act as channels for newly transcribed viral mRNA and, potentially, dsRNA (27). However, the limited resolution and lack of available homology models precluded building an atomic model of the viral capsid.

Considering the prevalence and limited understanding of TVV infection among the ubiquitous T. vaginalis, we sought to elucidate TVV structures using cryoEM and single-particle reconstruction techniques. The resulting cryoEM structure of TVV2 allowed us to characterize the structural features governing capsid assembly, mechanisms of genome sequestration, sites of mRNA release, and strategies of cytoplasmic maintenance of the viral mRNA. Additionally, comparisons of the TVV2 capsid with those of other dsRNA viruses featuring similar and distinct architectural characteristics offer insights into TVVs position in the evolutionary landscape of dsRNA viruses.

## RESULTS

### 3D reconstruction of TVV2 virions.

**FIG 1:**
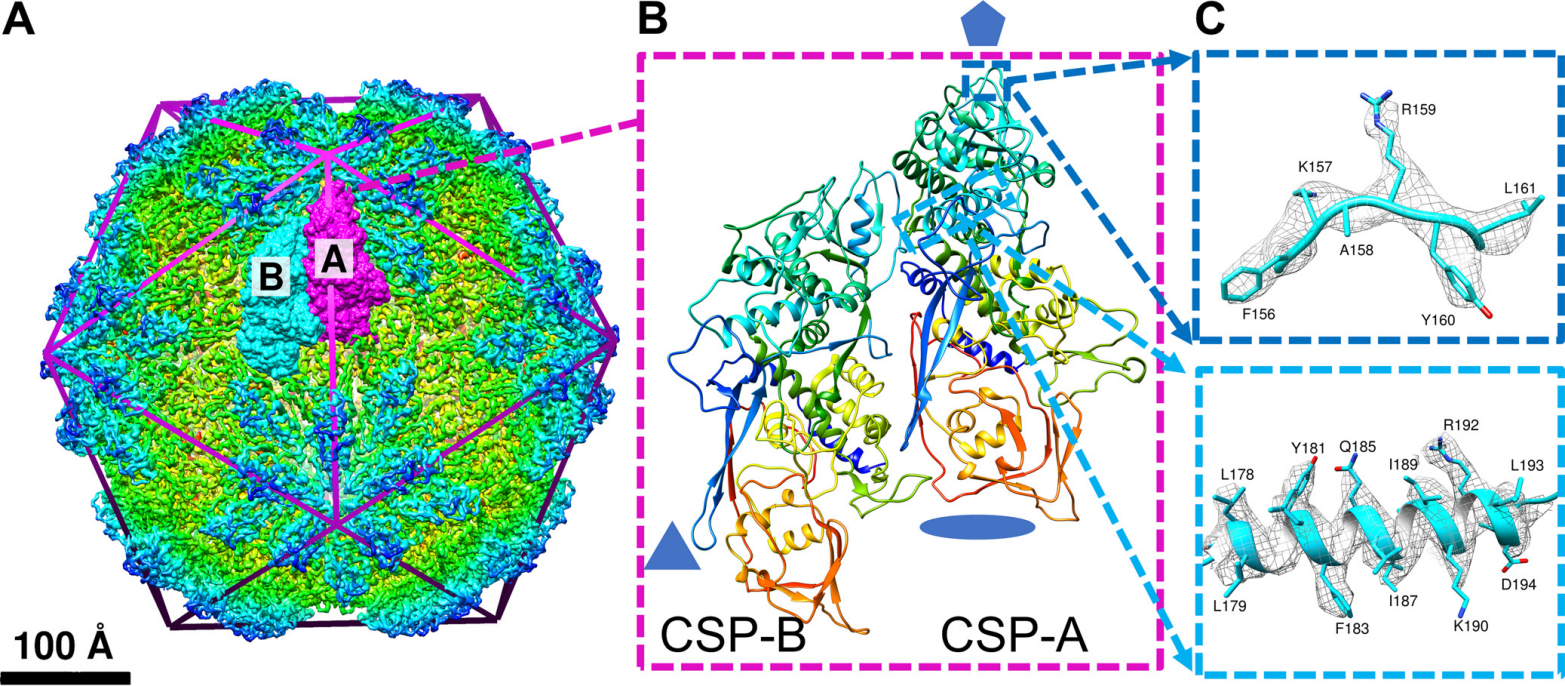
CryoEM reconstruction and atomic model of TVV2 capsid. (A) 4.0 Å cryoEM map of TVV2 icosahedral viral capsid, radially colored (rainbow) to enhance detail. Icosahedron edges are overlaid to indicate symmetry sites. Two conformers, CSP-A (magenta) and CSP-B (cyan), are displayed as surfaces fit into the cryoEM map. (B) Ribbon diagram of the conformer pair as shown in the cryoEM map, rainbow-colored from blue (N terminus) to red (C terminus). Residues preceding S37 and following P700 (CSP-A) and K701 (CSP-B) are not modeled. (C) Representative sample of the 3.6 Å subparticle map (gray mesh) with side chains of residues 156 to 161 (top, blue), and atomic structure of residues 177 to 196 (bottom, cyan) shown as fit.

TVV particles obtained from cellular subfractionation of Tv (strain G3) were imaged at a Titan Krios Electron microscope at 300 keV and nominal magnification of ×105,000 (see Materials and Methods below). From 2,034 micrographs, 2,493 particles were boxed and used for icosahedral reconstruction of the viral capsid to 4 Å (Fig. 1A and Fig. S1), based on the 0.143 “gold-standard” Fourier shell correlation coefficient (FSC) (28). Subparticle reconstruction about the I5 vertices improved the resolution of the decameric complex to ∼3.6 Å by the same FSC criteria (Fig. S1). Some flexible loops along the capsid exterior appeared less well-defined than the ordered helices of the CSP cores, but the arrangement of 10 CSPs surrounding the I5 vertex is consistent with the arrangement in other totiviruses (27, 29–31). Using the molecular modeling software Coot (32), and guided by secondary structural element predictions from Phyre2 (33), TVV2 CSP residues 37 to 700 were built into a single decamer subunit, denoted capsid shell protein conformer A (CSP-A), nearest the I5 vertex. CSP-A was used as a homology model for building residues 37 to 701 into the alternative subunit (CSP-B) situated further from the I5 vertices. This A-B pair constitutes the principle icosahedral asymmetric unit (IAU) of the viral capsid (Fig. 1A and B), and model quality can be observed based on fit in the cryoEM map (Fig. 1C).

10.1128/mBio.02924-20.1 FIG S1 Resolution assessment. (A) Local resolution evaluation on the whole virus icosahedral reconstruction (left) and the subparticle C5 reconstruction (right). (B) Fourier shell correction (FSC) coefficient as a function of spatial frequency (i.e., 1/resolution) for the whole virus icosahedral reconstruction and subparticle C5 reconstruction. Download FIG S1, TIF file, 6.4 MB. Copyright © 2021 Stevens et al. 2021 Stevens et al. https://creativecommons.org/licenses/by/4.0/ This content is distributed under the terms of the Creative Commons Attribution 4.0 International license.

### TVV2 capsid architecture and atomic models of the CSP conformers.

**FIG 2:**
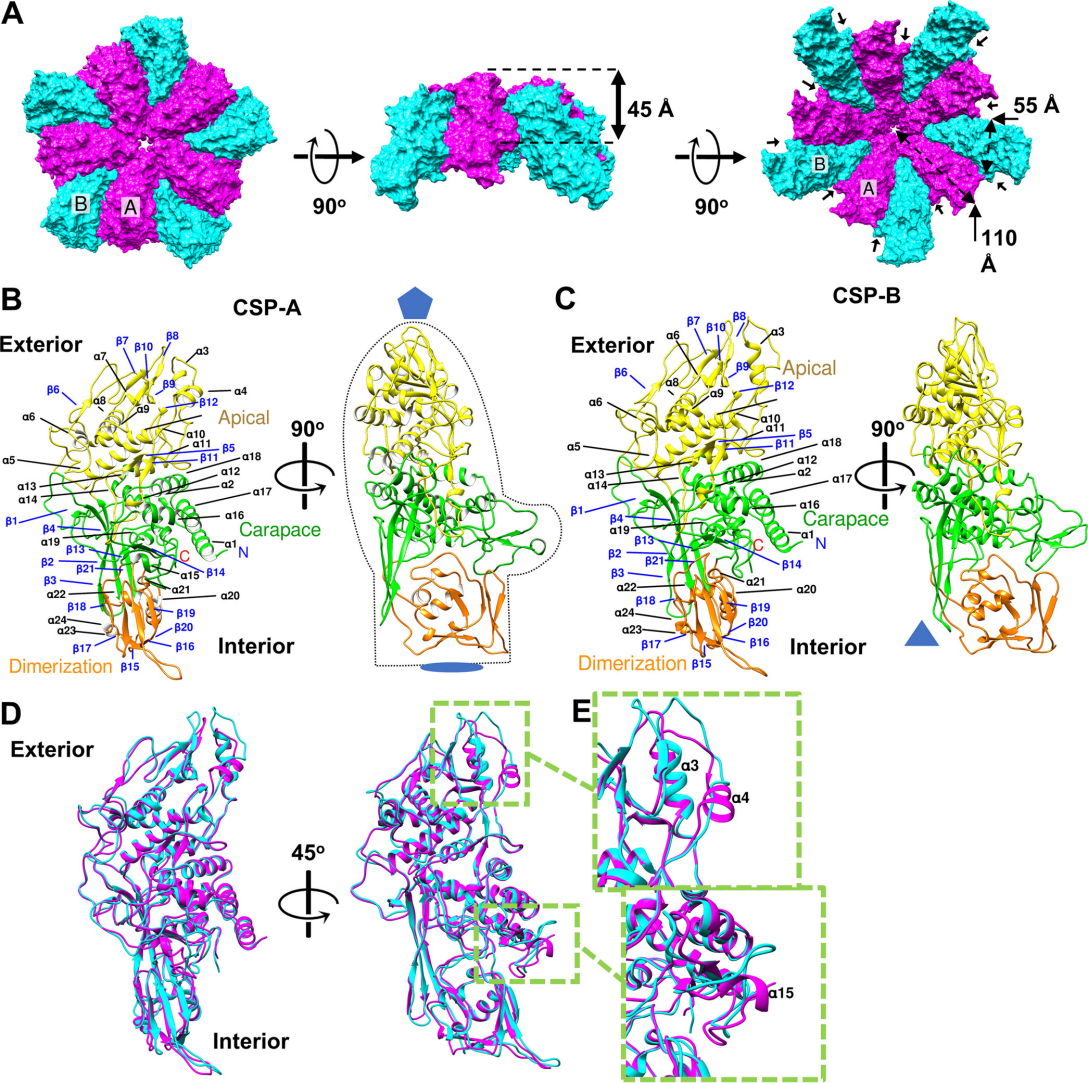
Characterization of the capsid shell protein. (A) Surface diagrams of decamers subunits with CSP-As (magenta) nearest the I5 vertex and surrounded by CSP-Bs (cyan) viewed from the exterior (left), side (middle), and interior (right). Approximate measurements of the conformer dimensions are overlaid in Å. Stabilizing protrusions are labeled with black pointers. (B and C) Orthogonal views of A and B conformers (CSP-A and CSP-B), respectively, with secondary structural elements labeled, symbols indicating sites of icosahedral symmetry, and mitten shape provided to enhance visualization. Colored: carapace, green; apical, yellow; dimerization domain, orange. (D) Ribbon diagram of CSP-A (magenta) and CSP-B (cyan) superimposed upon one another. (E) Close-up view of sites of structural discrepancies between conformers display on winding of α4 (top) and α15 (bottom) from CSP-A to CSP-B.

Like other totiviruses, the TVV2 viral capsid exhibits a T = 2* icosahedral capsid shell and measures ∼430 Å across at its widest near the I5 vertices, down to ∼350 Å at its narrowest between the icosahedral 2-fold (I2) symmetry axes. The TVV2 capsid is composed of 60 IAUs, organized into 12 decameric units centered about the I5 vertices, enclosing the viral genome. Within these decamers (Fig. 2A), five CSP-As are seated nearest the I5 vertex, crowding out five CSP-Bs which sit partially intercalated between the flanking CSP-As while extending between 5-fold axes to contact CSP-Bs from two other decamers forming the icosahedral 3-fold (I3) symmetry axes. Both conformers measure approximately 110 Å by 55 Å, with thicknesses varying from ∼45 Å near CSP centers to ∼20 Å near the I2 axes (Fig. 2A) and tertiary structures resembling the profile of a left-handed mitten (Fig. 2B), with the apical “finger” ends situated nearest the I5 vertices (Fig. 2B and C). Extending outward from the I5 vertices, the narrow “fingers” transition into the thicker “palm” and “thumb” before tapering to the thinner “cuff” end (Fig. 2B). This creates the appearance of raised plateaus extending outward along the I5 axes and sloping steeply toward the I2 and I3 axes approximately 70 Å from the I5 vertices (Fig. 2A). The decameric assembly of the monomers seems to grasp the flanking CSP between its “thumb” and “fingers” with separation between monomers creating the appearance of a grooved surface (Fig. 2A).

Following the naming conventions established for the inner capsid layer of bluetongue virus (BTV) (34), we likewise separated the TVV CSP into three domains. These domains encompass 24 α-helices (α1 to α24) and 21 β-strands (β1 to β21) and are denoted the apical “fingers” nearest the I5 vertices, carapace “palm” near the CSP centers, and dimerization “cuff” nearest the sites of I2 symmetry (Fig. 2B and C). While the boundary between the carapace and dimerization domains is clear, as the tertiary structure of the latter is appreciably thinner and proportionally richer in β-strands (Fig. 2B), the separation between the carapace and apical domains is not obvious. However, the amino acid sequence reveals an insertion whose tertiary structure is replete with short β-strands and loops seated atop the carapace without interweaving itself into other domains (Fig. S2).

**FIG 3:**
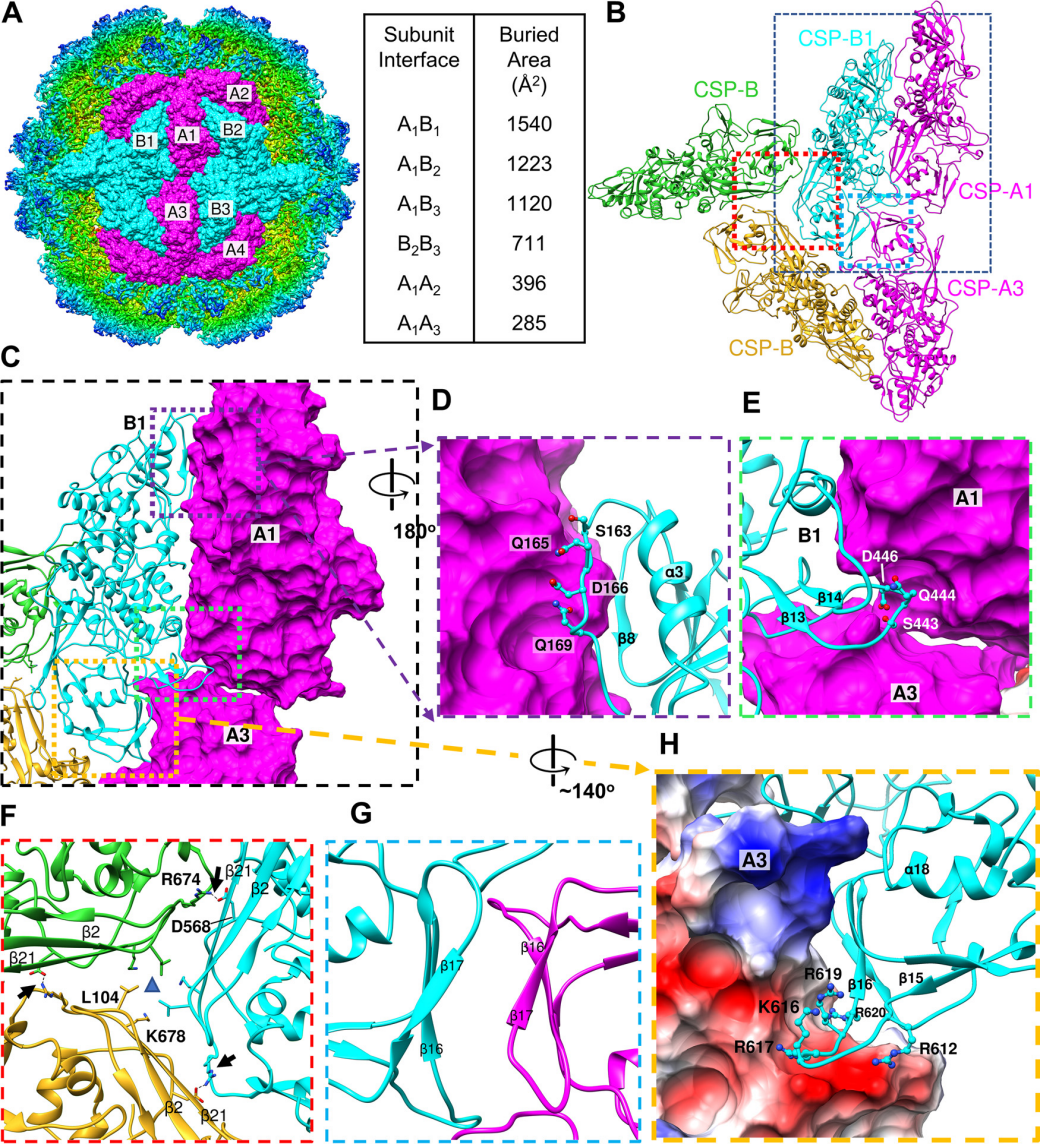
CSP interactions coordinate capsid structure. (A) Six copies of both CSP-A and CSP-B conformers fit into the TVV2 cryoEM map as viewed down the axis of I2 symmetry. Buried surface area (Å^2^) between subunit interfaces are listed at right. (B) Ribbon diagram of TVV capsid proteins illustrating contact surfaces between two CSP-As (magenta) and 3 CSP-Bs belonging to different decameric units (cyan, green, goldenrod). (C) Closer view of B_1_A_1_ interface with A_1_ and A_3_ surfaces displayed to illustrate contacts. (D) Close-up view of the unwound α4 of CSP-B with involved CSP-B residues shown. (E) View of the unwound α15 of CSP-B inserting beneath the long helices of the viral carapace domain. (F) View down 3-fold axis with residues involved in 3-fold symmetry position shown. (G) Opposing β-strands with β17 from both CSPs demonstrating slight twist toward β-sheet like character. (H) Electrostatic potential surface map displaying the positively charged residues of the β15-β16 loop of CSP-B, fit into the negatively charged (red) pocket between carapace and dimerization domains of CSP-A.

10.1128/mBio.02924-20.2 FIG S2 Sequence alignment of 4 recognized TVV strains and ScV-L-A illustrating disparate sequence identities. Secondary structural elements of TVV2 belong to the CSP-A conformer and are colored based on color scheme in Fig. 3B. Residues with 100% identity are boxed in red, and similar residues are bolded and boxed in yellow. Conserved residues from the I5 channel of TVV2 are marked with arrows (green) and those from the putative GTase site are marked with stars (red). Download FIG S2, TIF file, 5.2 MB. Copyright © 2021 Stevens et al. 2021 Stevens et al. https://creativecommons.org/licenses/by/4.0/ This content is distributed under the terms of the Creative Commons Attribution 4.0 International license.

The apical domain (residues 121 to 408) appears as an N-terminal insertion composed of 11 α-helices (α3 to α13) and 8 short β-strands (β5 to β12) (Fig. 2B and C, yellow). This domain exhibits a β-sheet sandwiched between 6 α-helices (α5 to α11) nearer the carapace domain and 2 α-helices (α3 to α4) which approach and surround the I5 vertices. Two loops between α3 to α4 and β7 to β8 extend polar residues (K157, R159, and N255) outward to line the I5 vertex generating the channels observed at the I5 axis (Fig. S2). Between α11 and α12, what appears to be an anchor loop, tipped with hydrophobic residues, extends over the core helices of the carapace toward the dimerization domain where it is encircled by residues of the latter. The involvement of the apical domain at both the I5 vertex and interface between carapace and dimerization domains suggests it may participate in varied functional roles.

The carapace domain (residues 37 to 120, 403 to 567, and 678 to 701) appears to act as the stable molecular core, composed of 8 α-helices and 7 β-strands concentrated around the molecule center (Fig. 2B and C, green). This domain contains a great number of the residues conserved across the TVV strains, along with many features implicated in the decameric subunit interface. Atop the structured core sit flexible loops that interact with and potentially stabilize adjacent domains and protrude from the CSP exterior forming the “thumb” of the conformers that insert into and stabilize the adjacent monomer (Fig. 2A). Several longer strands (β2 to β4) extend in a perpendicular manner from the helices of the carapace, forming a large antiparallel β-sheet which separates the dimerization domain from the I3 axes.

The dimerization domain (residues 568 to 677) is a C-terminal insertion composed of 5 helices (α19 to α23) and 6 strands (β15 to β20) which borders the carapace domain near the I2 and I3 positions and exhibits the thinnest section of the capsid shell (Fig. 2B and C, orange). The transition between the thick carapace and thin dimerization domains appears as a steep descent on the surface of the capsid creating a cleft lined with flexible coils originating from all three domains. The helices at the core of this domain are shorter than those observed in the others and appear sandwiched between β-sheets from both the dimerization and carapace domains. The 2 antiparallel β-sheets of this domain are conjoined perpendicularly to one another with a flexible loop protruding from the apex between β15 and β16.

CSP-Bs occupy a nonequivalent environment, further displaced from the I5 vertex compared to CSP-As, and introduce unique intersubunit interactions. CSP-Bs exhibits similar morphological structure to CSP-As, as illustrated by the limited differences when they are superposed atop one another (Fig. 2D). Analysis by the Dali server indicated a root mean square deviation (rmsd) of 1.4 Å calculated between all 664 aligned Cα atoms (Z-score = 47.7) of the pair (35). Key differences between the conformer structures are localized to sites of intersubunit contact (Fig. 2D), as the involved residues apparently fulfill different roles between conformers. Notably, we observe an unwinding of α4 and α15 from CSP-A to CSP-B to accommodate the interface with the adjacent CSP-A subunits (Fig. 2E).

### CSP-CSP interactions stabilize the TVV2 capsid.

The observed T = 2* organization of the TVV2 capsid is shared among many dsRNA viruses which vary in their size and complexity and incorporate differing numbers of capsid layers and stabilization strategies (36, 37). Regardless of the number of capsid layers or complexity, most dsRNA viruses employ interlocking domains such as the extensive N-terminal anchor or the auxiliary, “clamping” proteins of the single-shelled cytoplasmic polyhedrosis virus (CPV) or domain swapping of partiti-, chryso-, and quadriviruses (21–23, 38, 39). These interactions play an important role in maintaining the structural integrity of the viral capsid, concealing their dsRNA genomes from their hosts’ innate immune systems, and protecting those with extracellular phases from environmental stress (21–24, 38, 39). TVVs differ in these regards, as they have no extracellular phase and are not burdened by the robust antiviral machinery of higher-order eukaryotes during replication in protists. Instead TVVs must, at most, avoid detection by the hypothesized Tv RNAi system which passively detects exposed dsRNA genome (25, 26), raising the question as to whether TVVs must adhere to the same strategy for viral capsid assembly as their relatives in higher-order hosts.

Upon initial inspection, it was apparent that TVV capsid shells lacked any of the aforementioned interlocking domains of their distant cousins. In lieu of distinguishable CSP-CSP interlocking interactions, we surmised the order of TVV2 capsid assembly based on the buried surface area between CSPs (Fig. 3A and Movie S1). The CSP-pair, A_1_B_1_ (Fig. 1A and Fig. 3A), represents a reasonable initial species formed within the cytosol, as it buries the greatest surface area between subunits (∼1,540 Å^2^). The alternative A_1_B_2_ interface is ∼25% smaller (∼1,223 Å^2^), and both A_1_B_1_ and A_1_B_2_ have greater buried surface area than any other CSP-CSP interface (Fig. 3A). The apical and carapace domains appear solely responsible for the interactions governing intradecamer assembly, as the dimerization domains appear involved in anchoring the decamers together near the I2 positions. Interactions along the B_1_A_1_ interface (Fig. 3C) involve the unwound α4 from CSP-B_1_ which accommodates a protruding loop from the adjacent CSP-A_1_ subunit (Fig. 3D). Similarly, the unwound α15 of CSP-B_1_ inserts between the long β-strands (β2 to β4 and β21) of CSP-A_1_ and the dimerization domain of the CSP-A_3_ from the tetrameric IAU (Fig. 3E). These interactions are absent along the A_1_B_2_ interface wherein α4 is wound and involved in A_1_A_2_ interfaces, which likely coordinates assembly of IAUs into decamers but not monomers into IAUs. Likewise, the proximity of CSP-As to the I5 vertex renders α15 unable to insert beneath the long β-strands of CSP-Bs. Additionally, the interface between the possible tetrameric intermediate, A_1_B_1_-A_3_B_3_ (∼2,525 Å^2^), is ∼56% larger than the alternative A_1_B_1_-A_2_B_2_ tetramer (1,619 Å^2^) and ∼80% larger than the A_1_B_2_-A_4_B_3_ tetramer (∼1,405 Å^2^), suggesting that the first may be more stable and therefore more abundant in the cytosol (Fig. 3A). Interfaces at the I3 axes exhibit middling buried surface area between each monomer interface (∼711 Å^2^), but interactions appear localized to a protrusion (residues 671 to 674) beneath β21 of the adjacent CSP-B, indicating electrostatic interactions between R674 and D568 of neighboring monomers (Fig. 3F). The dimerization domains of the A and B subunits (Fig. 3A, CSP-A_3_ and CSP-B_1_) exhibit parallel β-sheets (β16, β17, and β19), with the slight twist within β17s creating the appearance of a near β-sheet if not for the approximately 6 Å separating them (Fig. 3G). The protruding loop from the floor of the dimerization domain (residues 610 to 620) appears rich in positively charged residues (R612, R617, K616, R619, and R620) and inserts into a negatively charged pocket between the carapace and dimerization domains of tetramer subunits (Fig. 3H). This insertion may act as an anchor to stabilize the association of the capsid decamers through electrostatic interactions, protecting the viral genome in place of more robust interlocking strategies. Based on our observations, it appears that TVV2 employs a distinct strategy to assemble and stabilize the capsid in a manner optimized to conceal the dsRNA genome in the homeostatic cytosolic environment.

10.1128/mBio.02924-20.3 MOVIE S1 External view of TVV2 decamer provided to orient viewer above I5 axis. Overlay of surface diagram colored based upon hydrophobic character with orange representing hydrophobic and gray-blue being hydrophilic. Hydrophobic patches are observed along the decameric interfaces along each CSP. Download Movie S1, MPG file, 16.4 MB. Copyright © 2021 Stevens et al. 2021 Stevens et al. https://creativecommons.org/licenses/by/4.0/ This content is distributed under the terms of the Creative Commons Attribution 4.0 International license.

### TVV2 capsids organize and coordinate the viral genome.

**FIG 4:**
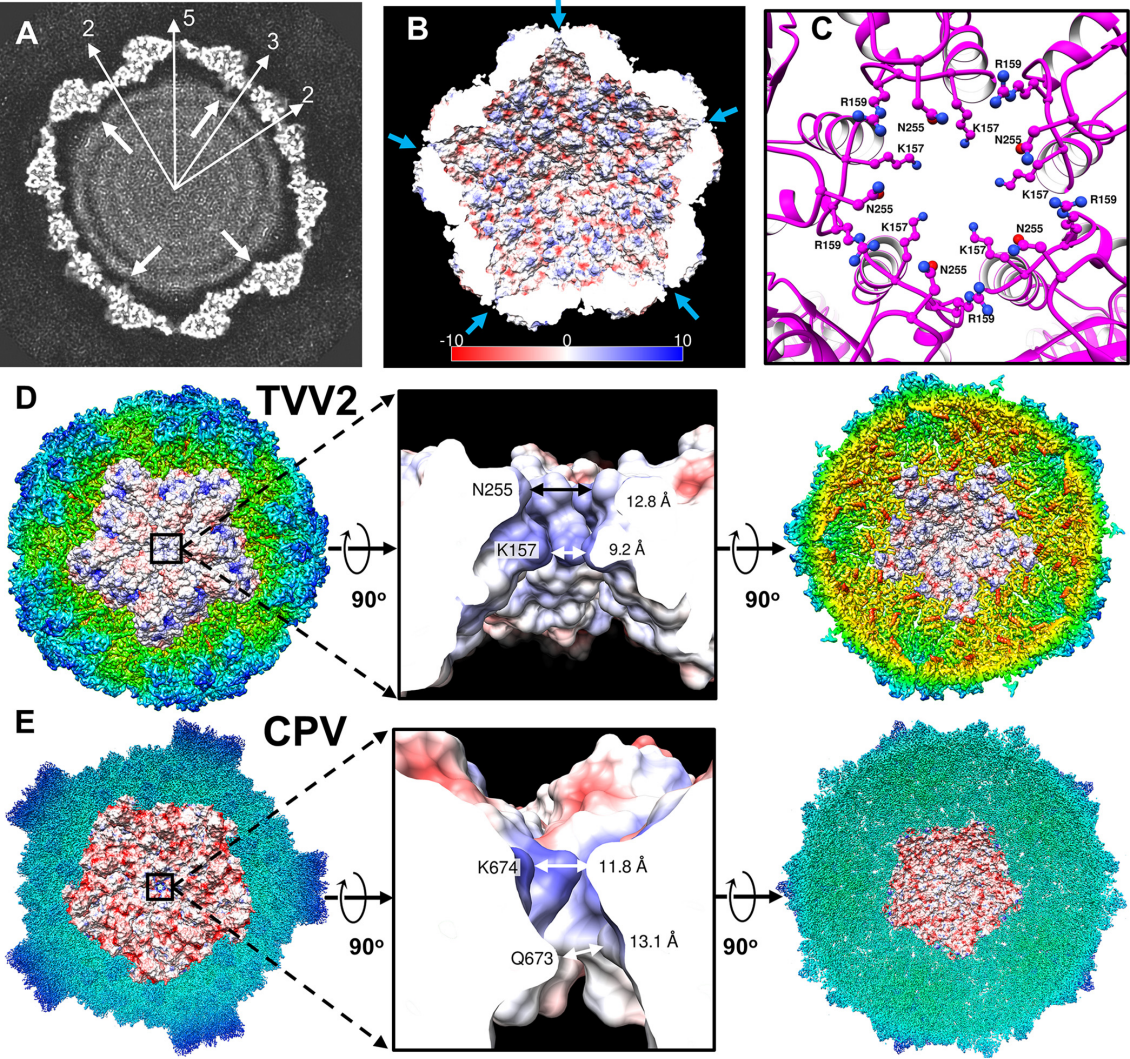
TVV2 capsid coordinates genome arrangement and egression of transcription intermediates. (A) 10 Å thick slab view of the Icosahedral viral capsid of arbitrary contour colored in grayscale with map densities in white and 0-values in black. Sites of icosahedral symmetry through the slab are labeled with thin, white arrows, and thick, white arrows indicate the location of the C terminus based on the atomic model. Distance between dsRNA strands measures ∼30 Å. (B) Cutaway view of capsid interior with electrostatic potential diagrams illustrating positive (blue) and negative (red) surfaces. I5 vertices are marked with arrows (blue). (C) I5 vertex as viewed from the capsid exterior; CSP-A K157, R159, and N255 residues are shown as ball and stick diagrams. (D) Exterior view of the TVV2 capsid protein decamer fit into the icosahedral map (radially colored) with surface colored by electrostatic potential (left). 90^°^ rotation of A and cutaway view of the 5-fold channel, with local residues from a CSP-A subunit (K157, R159, and N255) and channel diameters labeled (middle). Interior view of TVV2 decamer surface fit into map (right). (E) Exterior view of the dsRNA virus CPV CSP in the quiescent state (RCSB: 3JAZ) decamer fit into the icosahedral map with surface colored based on electrostatic potential (left). A cutaway view of the 5-fold channel, with involved residues from an CSP-A conformer (K674 and Q673) and channel diameters labeled (middle). Interior view of CPV CSP decamer fit into map (right).

In contrast to the grooved exterior of TVV2 capsids, the interior appears smooth, as demonstrated in a cross section of the capsid at an arbitrarily high contour level (Fig. 4A). This planar view also reveals three concentric ring-like densities, likely corresponding to the dsRNA genome, featuring an approximate interduplex distance of 33 Å (Fig. 4A), slightly greater than those of other dsRNA viral genomes which measure around 27 to 30 Å (40, 41). The outermost ring of the TVV2 genome appears traced along the interior surface of the capsid in a quasi-hexagonal arrangement, separated from the interior wall by as little as ∼10 Å below the I2 positions and up to ∼30 Å at the I5 vertices (Fig. 4A). In addition to increased separation, the outer ring of the dsRNA genome exhibits perturbations beneath the I5 vertices, suggesting that a structure poorly conserved during icosahedral averaging occupies this space. The location of the C-terminal tail of CSP, thought fused to the RdRp complex, sits adjacent to the negative space beneath the I5 portal (Fig. 4A, white arrows).

The capsids of dsRNA viruses frequently exhibit electronegative interiors, hypothesized to improve transcriptional efficiency among other dsRNA viruses of low genomic densities (36, 42). Electrostatic potential maps of the atomic model revealed the varied charge properties of the TVV2 capsid (Fig. 4B). The interior capsid surface appears predominantly electronegative (red) with discrete electropositive regions (blue) including the protruding loop of the dimerization domain and residues along α1 nearer the I5 vertices (Fig. 4B). Additionally, secondary structure predictions of the 36 unmodeled N-terminal CSP residues indicate a flexible domain with a calculated isoelectric point of 3.8. The position of α1 along the CSP interior suggests that this flexible domain may extend inward and provide greater negative character along the capsid’s inner surface. These charge properties are reminiscent of those observed along the interior of fungal dsRNA viruses in addition to picobirnaviruses wherein these interactions are hypothesized to prevent the association of the genome with the capsid surface via electrostatic repulsion of the negatively charged phosphate backbone (36, 42). This phenomenon, combined with the lower genomic density of TVV relative to that of other dsRNA viruses (Table 1), likely explains the consistent separation observed between the capsid and template and contributes to increased template motion as reflected by the poorly resolved nature of the dsRNA genome (Fig. 4A).

**TABLE 1:** Genome packaging densities of double-stranded RNA (dsRNA) viruses^*a*^

Virus		Genome features			Capsid features		
Family	Member	No. genome segments	Size (kbp)^*c*^	Length (μm)^*d*^	CSP (aa’s)	Φ^*e*^/ir^*f*^ (nm)	Genome density (bp/100 nm^3^)^*g*^
*Herpesviridae*	HCMV^*b*^	1	200	68	1,370	130/45	38
*Reoviridae*	*Cypovirus*	10	31.4	8.79	1,333	58/24	54
	*Aquareovirus*, GCRV	11	23.6	6.63	1,027	60/23	46
	*Orbivirus*, BTV	10	19.2	5.4	901	52/22	43
	*Rotavirus*	11	18.5	5.18	880	52/23.5	34
*Cystoviridae*	Phage Φ6	3	13.4	3.77	769	50/20	40
*Totiviridae*	TVV2	1	4.65	1.31	709	43/18	19
	ScV-L-A	1	4.6	1.29	680	43/17	21
*Picobirnaviridae*	2	4.2	1.18	590	35/14	36
*Partitiviridae*	PsV-S^*h*^	1(2)^*i*^	1.7/3.3	0.477	420	35/12	23
*Chrysoviridae*	PcV^*h*^	1(4)^*i*^	3.2/12.6	0.899	109	40/16	19
*Quadriviridae*	RnQV1	1–2(4)	4.3/17.1	1.21	1,356 + 1,061	4,716	25 (50)^*j*^

a Table adapted with data from Luque et al. (36).

b Human Cytomegalovirus, a dsDNA virus, a lineage lacking genome segmentation, and characterized with B-from dsDNA.

c Approximate genome length.

d Genome length calculated on rise/bp of 2.81 Å for dsRNa and 3.4 Å for dsDNA.

e Approximate outer diameter.

f Approximate inner radius.

g Genome density when capsid cavity is assumed to be a perfect sphere containing only nucleic acids.

h PsV-S, penicillium stoloniferum virus S; PcV, penicillium chrysogenum virus.

i PsV-S and PcV have genomes formed of 2 and 4 segments, respectively.

j Density value of 25 if there is 1 dsRNA molecule per particle or 50 if there are 2 dsRNA molecules per particle.

As dsRNA viruses must export transcription intermediates into the cytosol, we sought to locate a site capable of such activity. As noted in the planar view, channels spanning the width of the capsid shell are located at the I5 vertices (Fig. 4A). These I5 channels are lined with residues extending from loops between α3 to α4 and β7 to β8 within the apical domain. Closer inspection of the decamer model from the outside reveals the residues lysine 157, arginine 159, and asparagine 255 from all 5 CSP-As lining this channel (Fig. 4C). A cutaway view through the electrostatic potential map of the I5 vertex illustrates the positively charged nature of this channel (Fig. 4D). The channel measures approximately 9.2 Å in diameter nearest the capsid interior, to 12.8 Å near the exterior, and this contrasts with the 16 to 20 Å I5 channel of TVV1 derived from a lower-resolution cryoEM map in a previous investigation (Fig. 4D) (27). The internal diameter of the TVV2 I5 channel may be expanded to approximately 10.8 Å via conformational rearrangement of K157 residues, providing a reasonable egression site for viral mRNA. Sequence alignment of the major TVV strains (TVV1 to 4) indicates that K157, R159, and N255 residues are conserved despite divergent sequences, suggesting that these residues may be necessary for channel functionality (Fig. S2). Structural comparison of the TVV2 channel to that of CPV from *Reoviridae* reveals shared features, including similar channel diameters of 11.8 to 13.1 Å and linings of positively charged residues (Fig. 4D and E), despite significantly different capsid size and complexity (43).

### Putative guanylyltransferase sites on the TVV2 capsid exterior.

**FIG 5:**
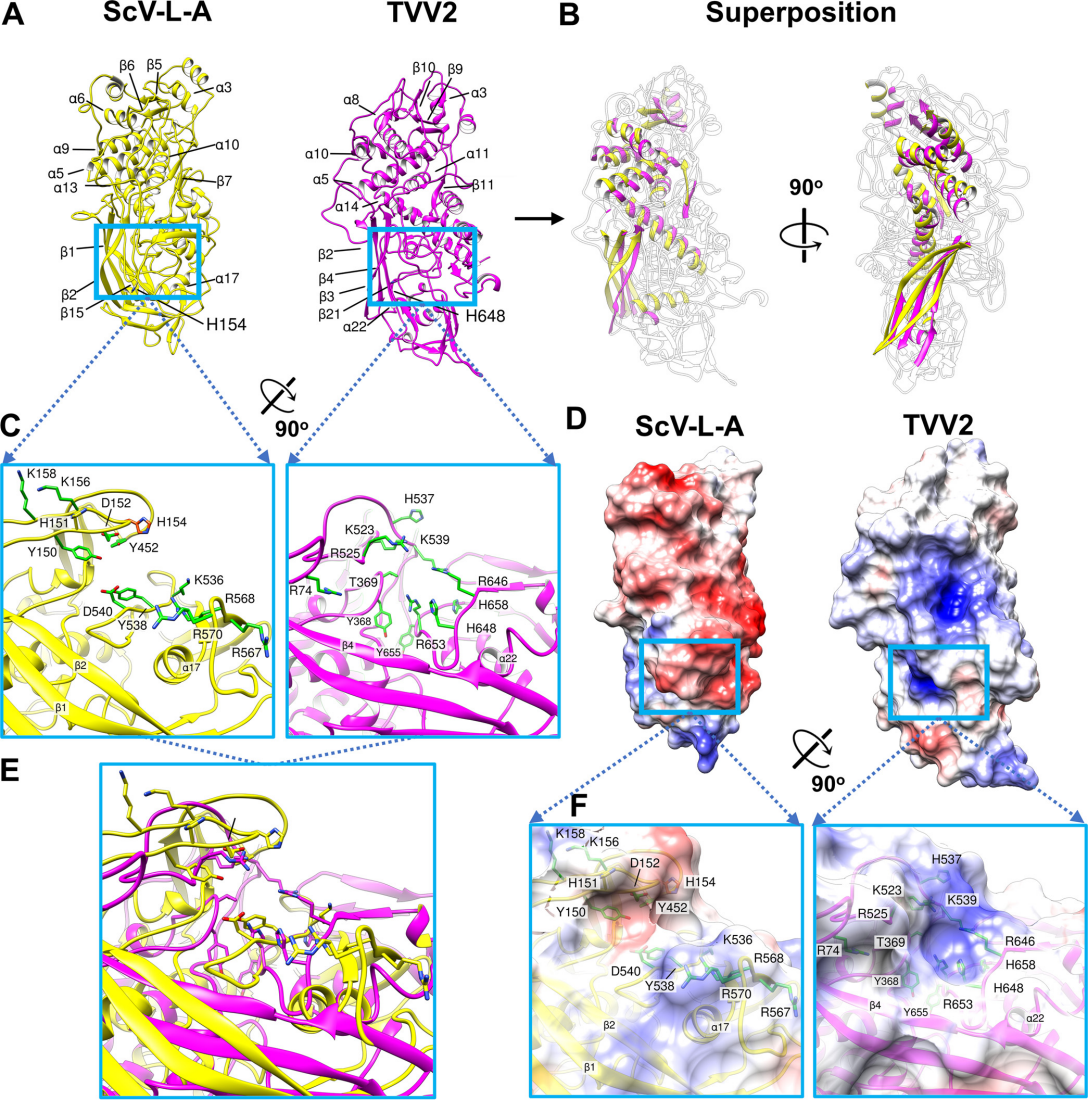
Structural comparison of TVV2 CSP to ScV-L-A Gag. (A) Exterior view of ScV-L-A (yellow) and TVV2 (magenta) capsid shell proteins displayed with secondary structural elements of similar position labeled. (B) Orthogonal view of similar secondary structural elements between TVV2 and ScV-L-A capsid shell proteins superposed with outlines of the nonconserved segments. (C) Zoomed in view of ScV-L-A GTase site colored in yellow (left) with residues implicated in enzymatic activity (Y150, D152, K156, K158 Y452, D540, Y538, K536, R567, R568, and R570) colored green and the essential His154 labeled in orange (left). The labeled and putative GTase site of TVV2 is colored in magenta (right) with local residues colored green and labeled (R74, Y368, T369, K523, R525, H537, K539, R646, H648, R653, Y655, and H658) (right). (D) Exterior view of electrostatic potential maps of ScV-L-A (left) and TVV2 (right) CSP monomers, with negative (red) and positive (blue) charge distributions. Opacity changed to illustrate residue locations. (E) Enzymatic sites from panel C superposed. (F) Close-up views of the sites from panel C with surfaces colored based on electrostatic potential.

The global architecture of TVV2 capsids is reminiscent of the well-characterized L-A virus of Saccharomyces cerevisiae (ScV-L-A) (29). Both viruses encode a single capsid protein, 120 copies of which encapsidate unsegmented dsRNA genomes (15, 44). Although they share little sequence identity (<19%) (Fig. S2), both TVV2 and ScV-L-A virions exhibit T = 2* icosahedral symmetry and similar α-helix-rich folds (Dali server reported an rmsd of 4.7 Å across 365 aligned Cα residues [Z-score = 7.8] of the two proteins). In particular, superposition of the two proteins reveals that 5 α-helices and 7 β-strands are shared between the molecule cores (Fig. 5A and B). Moreover, comparison of the well-characterized guanylyltransferase (GTase) site of Gag to the corresponding position along CSP-A revealed a cleft with similar amino acid composition, including amino acids integral to ScV-L-A GTase activity (Fig. 5C and E) (29, 45). In the TVV2 CSP-A we observe positively charged residues (R74, K523, R525, K539, R646, and R653) arranged on the cleft exterior generating a positively charged region similar to that observed in ScV-L-A (Fig. 5D and F). Likewise, Histidine residues (H537, H648, and H658) line the cleft interior while the aromatics (Y368 and Y655) appear buried deeper within the cavity (Fig. 5C and E). In a similar manner to that of the ScV-L-A GTase site, the local residues in the CSP cleft are directed toward a central point, indicating where they may interact with host mRNA and coordinate the enzymatic capture of the 5′ mRNA cap (Fig. 5C and E). However, the precise positioning of these amino acids differs between the two viruses, including the position of histidine residues which are integral for cap cleavage in ScV-L-A, wherein they form the cap-His intermediate that precedes cap transfer to viral mRNA (Fig. 5C and E) (29, 45, 46).

## DISCUSSION

Among totiviruses, the single capsid layer is responsible for protecting and sequestering the viral genome while facilitating RdRp activity and the egression of viral mRNA into the cytosol (27, 29–31). The details revealed by our 3.6 Å structure of the TVV2 capsid represent a marked improvement over other protozoan totivirus capsid structures, which had previously been limited to ∼6.0 Å resolution (27, 31), insufficient to build atomic models. Importantly, the TVV2 atomic model we derived enables us to characterize the interactions among its capsid subunits and draw meaningful insights from the features governing capsid assembly and maintenance of the viral genome.

The proposed assembly pathways of icosahedral dsRNA viruses featuring T = 2* capsids have historically belonged to two camps, both of which begin with the formation of the basic asymmetric unit (AU), 60 of which are used to generate the capsid (47). The first pathway, often observed among *Reoviridae*, relies on the formation of compact decamers composed of 5 AUs surrounding the I5 axes, 12 of which combine to form the complete capsid (34, 47–49). The second pathway involves the formation of tetramers, composed of AU dimers and related about the I2-symmetry axis, which has been suggested for many single-layered dsRNA viruses (21, 50, 51). In the first pathway, absent nucleating factors, the formation of decameric intermediates is kinetically disfavored, as the incomplete decamers would have low concentration and subsequent combinations of these complexes would be similarly impeded by slow diffusion and low concentration (51). In contrast, smaller, I2-related tetramers represent far more kinetically favorable intermediates, likely existing in much higher concentrations in the cytosol and functioning to conjoin neighboring decameric complexes. In the case of TVV2, we observe what may be the strongest complementary electrostatic interactions between the I2-related CSPs, with positively charged protrusions inserting into negatively charged pockets of the I2-related CSPs (Fig. 3H). This, in addition to the significantly greater buried surface area between the A_1_B_1_-A_3_B_3_ tetramer than that between the alternative tetramers, suggests that A_1_B_1_-A_3_B_3_ represents the most stable and therefore most abundant capsid intermediate.

Unlike many dsRNA viruses, most totiviruses, including TVV2, do not transmit extracellularly and are instead transmitted vertically from mother to daughter cells, similar to plasmid inheritance (27, 52–55). However, the dsRNA genomes of these viruses present distinct challenges to replication which differentiate them from other extranuclear genetic material. As dsRNA is a potent inducer of eukaryotic antiviral response (24, 56), the viral genome must be enclosed within a protective capsid, necessitating dsRNA-specific replication strategies including transcription via capsid-associated RdRp complexes and coordinated maintenance of these transcription intermediates (27, 29–31). The TVV2 capsid appears to closely conform to the general morphology of previously characterized totiviruses and is similarly devoid of any features indicating extracellular transmission. As these capsids do not encounter the robust immune system found in higher-order eukaryotes or the harsh conditions of the extracellular environment, it is conceivable that the strategies governing capsid assembly and stability may vary greatly from those that do. Other dsRNA families such as *Chrysoviridae*, *Partitiviridae*, *Quadriviridae*, and the well-characterized *Reoviridae* include multi- and single-layered viruses transmitted both extracellularly and intracellularly across hosts of various complexities. Regardless of layers or mode of transmission, all of these viruses incorporate specific domains that buttress the association between the subunits of their icosahedral T = 2* capsid shells (21–23, 38, 39, 57, 58). The TVV2 capsid displays similar T = 2* symmetry but does not exhibit comparable subunit interactions and instead seems to rely on the abutting interface between the CSP conformers. The TVV CSPs are thicker (∼45 Å) than those of the reoviruses BTV (∼35 Å) and CPV (∼30 Å) (34, 39), and this seemingly provides ample lateral contact area between TVV subunits (Movie S1). Interestingly, those dsRNA viruses featuring capsid stabilizing domains infect hosts whose innate immune systems utilize pattern-recognition receptors (PRRs), whereas totiviruses infecting hosts lacking PRRs, including ScV-L-A and now TVV2, appear to lack such domains (25, 26, 59, 60). Additionally, TVV capsids have been shown to be more prone to degradation under conditions of increased temperature or pH than the extracellularly transmitted totivirus infecting Giardia lamblia, a host which may employ similar antiviral RNAi defenses to Tv (26, 31). Along with our observations of the associations between TVV2 subunits, this indicates that these less-robust interactions are likely sufficient to conceal the viral genome from the passive RNAi pathways and maintain capsid integrity within but not outside the cytosol. This also suggests that the robust stabilizing strategies common among dsRNA viruses of higher-order eukaryotes are not necessary for efficient replication in their protozoan bound relatives.

As the dsRNA genome remains enclosed within the TVV capsid throughout its replication cycle, RdRp complexes are necessary to transcribe viral mRNA for translation at the host ribosomes. Further, it has been demonstrated that RdRp activity in other dsRNA virus families such as *Reoviridae* is dependent on encapsidation for transcriptional activity (61–63). It is possible, therefore, that TVV RdRp activity may be likewise dependent on the capsid, with the shell perhaps facilitating RdRp-genome interaction and transcription. In the case of ScV-L-A, it is thought that the translational efficiency of the −1 ribosomal frameshift leads to the incorporation of just 1 to 2 RdRps per capsid (64). The RdRp of TVV2 is likewise thought to be expressed as a minor, C-terminal fusion protein to the CSP via −1 ribosomal frameshifting (15, 58, 65); however, the RdRp stoichiometry has not been firmly established. The limited number of RdRp complexes means that even when data sufficient for a high-resolution reconstruction of the viral capsid are available, there are often too few RdRp complexes to resolve a structure. Indeed, despite extensive efforts following previous strategies to resolve asymmetrically attached structures (61, 66), we were unable to resolve any density corresponding to the RdRp complex. Further effort with a substantially larger number of images and an optimized image processing method is needed to locate and resolve the RdRp within TVV2. Notably, we observe the TVV CSP C terminus, to which the RdRp is fused (15), beneath the I5 vertices and adjacent to the only visibly unoccupied space of reasonable size to accommodate the 83 kDa RdRp domain (Fig. 4A).

Consistent with other dsRNA viruses, like those in the *Reoviridae* family (61, 66), our data suggest the placement of the RdRp complexes beneath the I5 vertices, and this may provide nascent viral mRNA direct access to the I5 vertex. As previously mentioned, the I5 channel is the largest capsid spanning feature of TVV2, measuring 9.2 to 12.8 Å across, making it the most reasonable site for mRNA egress. This diameter is significantly smaller than the 16 to 20 Å suggested in previous findings of TVV1 (27); however, the positively charged residues lining the I5 channel in TVV2 appear conserved across TVV1-4, suggesting this feature may be present throughout TVVs (Fig. S2). Interestingly, the channel dimensions are consistent with those of distantly related *Reoviridae* members like CPV and are significantly smaller than the 18 Å openings at the I5 vertices of ScV-L-A (29, 43). During transcription of viral mRNA in TVV2, we suspect that the positively charged residues at the I5 channels act as attractants for the negatively charged phosphate backbone of the mRNA to coordinate its transport into the cytosol. It is conceivable that the proximity of these channels to the newly synthesized mRNA benefits export by limiting the opportunity for viral mRNA to adopt secondary structures within the capsid, further supporting our assignment of RdRp beneath the I5 vertices.

Though the capsid and RdRp complex are capable of synthesizing and transporting the viral mRNA to the cytosol, TVV’s inability to synthesize a 5′ cap leaves the mRNA susceptible to detection and degradation by the exoribonucleases of the host cell (67, 68). As such, an enzymatic GTase domain capable of scavenging the 5′ caps from host mRNA and transferring them onto viral mRNA must be incorporated into the limited TVV2 proteome to ensure efficient translation. As the CSP is the only TVV2 protein with apparent access to cytosolic mRNA, the cap-snatching domain is very likely incorporated here. Structural comparison between the TVV2 CSP and Gag of ScV-L-A revealed similar α-helix-rich folds and analogous histidine rich sites along their exterior surfaces (Fig. 5). These similarities suggest that TVV2 may employ a comparable cap-snatching strategy with species-specific variations. In TVV, the putative enzymatic cleft is less pronounced than the trench of ScV-L-A, but similar positively charged residues lining the exterior may nonetheless assist in attracting and coordinating the negatively charged phosphate backbone of host mRNAs. Aromatic residues within the putative enzymatic cleft (Y368 and Y655) may stabilize the guanosine ring of the host mRNA’s 5′ cap via π-π stacking interactions similar to those of other GTase sites (29, 43). The presence of multiple histidine residues suggests that the putative TVV2 site may carry out the nucleophilic attack of the cap’s phosphate backbone to generate a cap-His intermediate in a similar manner to that of ScV-L-A (45). Interestingly, despite low sequence identity, Clustal alignment revealed that many of the residues in this region, including one histidine (H648), are conserved across all TVV strains (Fig. S2), suggesting that these residues may be important to TVV replication. Having 120 highly similar CSPs containing a GTase domain may enable the decapitation of many times more host mRNA than are needed to provide caps for the nascent viral mRNA. As has been suggested for ScV-L-A (69), these decapitated host mRNAs may serve as decoys to distract the host’s mRNA degradative machinery preceding translocation of the vulnerable, uncapped viral mRNA. Likewise, the many 5′ caps decorating the capsid surface may give the viral mRNA more opportunities to acquire a cap before traveling to the ribosome. It is conceivable that such cap-snatching activity may also induce conformational changes within CSPs which initiate RdRp activity and/or expand the I5 channels providing transcriptional regulation.

If the cap transfer reaction catalyzed by the putative GTase of TVVs is reversible and somewhat promiscuous, TVV superinfection may prove mutually beneficial to the viruses. Here, the capacity of the putative TVV GTase domain to transfer caps nonspecifically between viral mRNAs would enable caps procured by one TVV strain to be transferred to the mRNA of another, helping to establish superinfection. Though we cannot confidently assert the identity or strategy of the TVV cap-snatching domain at this time, the suggested scenario represents an exciting opportunity through which future biochemical and structural investigations may uncover a cap-snatching strategy shared among totiviruses.

## MATERIALS AND METHODS

### Virus preparation.

T. vaginalis strain G3 was cultured in Diamond’s modified Trypticase-yeast extract-maltose (TYM) medium supplemented with 10% horse serum (Sigma-Aldrich), 10 U/ml penicillin,10 μg/ml streptomycin (Gibco), 180 μM ferrous ammonium sulfate, and 28 μM sulfosalicylic acid (70). Parasites, grown at 37°C and passaged daily, were harvested by centrifugation and washed twice with phosphate-buffered saline. The cells were resuspended in 50 ml buffer 1 (2% IGEPAL CA-630, 2% Triton X-100, 10% glycerol, 10 mM Tris, 2 mM EDTA, 150 mM KCl, 2 mM MgSO_4_, 1 mM dithiothreitol [DTT], 1× Halt protease inhibitors [pH 7.4]) and lysed in a Stansted cell disrupter with 30 lb/in^2^ front pressure and 12 lb/in^2^ back pressure. The cell lysate was centrifuged at 1,000 × *g* for 10 min, and the resulting supernatant was further centrifuged at 5,000 × *g* for 30 min to obtain the pellet enriched with TVV particles. The pellet was resuspended in buffer 2 (150 mM NaCl, 50 mM Tris, 2 mM MgCl_2_, 1 mM DTT, 1× Halt protease inhibitors) and was found to contain TVV particles based on cryoEM examination.

For cryoEM sample preparation, 2.0 μl of the sample was applied to a glow-discharged lacey grid (Ted Pella) and incubated for 1 min. The grid was then washed once with buffer 2 to remove large debris on the grid. Approximately 1.5 μl of buffer remained on the grid before being transferred into a manual plunger apparatus. The grid was then blotted manually and flash-frozen in liquid ethane. The frozen grids were stored in liquid nitrogen prior to cryoEM imaging.

### CryoEM imaging.

Movies of dose-fractionated image frames were acquired in a Titan Krios microscope (Thermo Fisher Scientific) equipped with a Gatan imaging filter (GIF) Quantum LS and a post-GIF Gatan K2 Summit direct electron detector operated in superresolution mode at a nominal magnification of 105,000× (yielding a calibrated pixel size of 0.68 Å on the sample level) with SerialEM (71). The GIF slit width was set to 20 eV. The dose rate on the camera was set to ∼8 electrons/pixel/s, and the total exposure time of each movie was 6 s, which fractionated into 30 frames of images with 0.2 s exposure time for each frame. Dose-fractionated frames were 2× binned (pixel size 1.36 Å) and aligned for beam-induced drift correction to generate both dose-weighted (used for final reconstruction) and dose-unweighted (used for manual screening, contrast transfer function (CTF) determination and particle picking) averaged micrographs using MotionCorr2 (72).

### Data processing.

The defocus values of the micrographs were determined with CTFFIND4 (73). Micrographs with ice contamination or defocus value outside the range −0.8 to −3 μm were discarded. From a total of 2,177 micrographs, 2,034 were selected, and 5,076 TVV manually picked particles were boxed out in 480 square pixels and 2× binned to 240 square pixels (pixel size of 2.72 Å) to speed up further data processing with RELION 3.1 (74). Orientation and center parameters of each particle image were first determined and refined with icosahedral symmetry, yielding a 5.6 Å reconstruction. After one round of 3D classification, 2,493 high-quality particle images (49% of all particles) were selected and reextracted by centering the coordinates for further refinement. Then, these high-quality particles were subjected to another round of 3D refinement with icosahedral symmetry, yielding a 4.0 Å reconstruction (Fig. S1) finally with the benefit of CTF refinement.

To obtain a higher resolution reconstruction for model building, we used a subparticle reconstruction strategy similar to those detailed in previously published papers (61, 66, 75). Briefly, the RELION command “relion_particle_symmetry_expand” was used with the icosahedral symmetry option (I3) to expand the icosahedral orientation entry in the data file generated from icosahedral reconstruction step into 60 icosahedrally oriented entries in a new data STAR file. In these 60 entries, every five C5 related entries corresponded to one penton vertex, so only one entry of those five was kept, yielding a duplicated-entries-removed data STAR file. The icosahedral reconstruction (I3) orients in such a way that one of its penton vertices is in the positive *z* axis so we could easily estimate its coordinates (for example, *x* = 0, *y* = 0, and *z* = 110 pixels in the bin1 map) in the map. The coordinates for this penton vertex and the duplicated-entries-removed data STAR file were then used as the input to reextract 12 penton vertex subparticles from every TVV virus particle image (29,916 subparticles in total). By imposing C5 symmetry, RELION further refined the local orientation and center parameters of these vertex subparticles, and we obtained a reconstruction of penton vertex capsid with an average resolution of 3.6 Å (Fig. S1).

The resolution of the cryoEM maps were estimated on the basis of the 0.143 gold-standard FSC criterion (Fig. S1) (28). The cryoEM maps were sharpened with a B-factor (−80 Å^2^) in RELION. The local resolution evaluations were determined by ResMap (76).

### Atomic modeling, model refinement, and graphics visualization.

Based on the positions of discernible aromatic residues and guided by secondary structural predictions from Phyre2, TVV2 was identified as the resolved structure during *de novo* atomic model building in the macromolecular modeling software Coot (32, 33). The resulting model was improved with PHENIX following the procedure detailed previously (77). The models were then refined by the PHENIX real-space refine function and validated by the worldwide protein data bank (wwPDB) validation server (78). Visualization of the atomic model, including figures and movies, are made using UCSF Chimera (79). Sequence alignments were made using Clustal Omega and alignment visualizations were rendered using ESPript 3.0 (80, 81). Structural comparisons between capsid shell protein conformers were quantified using the Dali server (35). Buried surface area between capsid subunits was calculated using ChimeraX “measure buriedArea” function (82).

### Data availability.

The cryoEM density maps and atomic models are deposited in the Electron Microscopy Data Bank and the Protein Data Bank under the accession codes EMD-23560, 7LWY and EMD-23561, 7M12 for the focused and complete assemblies, respectively.
