# Supplementary figures and images for: Atomic Structure of the Trichomonas vaginalis Double-Stranded RNA Virus 2
Source: mBio. 2021 Mar 30;12(2):e02924-20. doi: 10.1128/mBio.02924-20 (PMC8092272; doi:10.1128/mBio.02924-20)

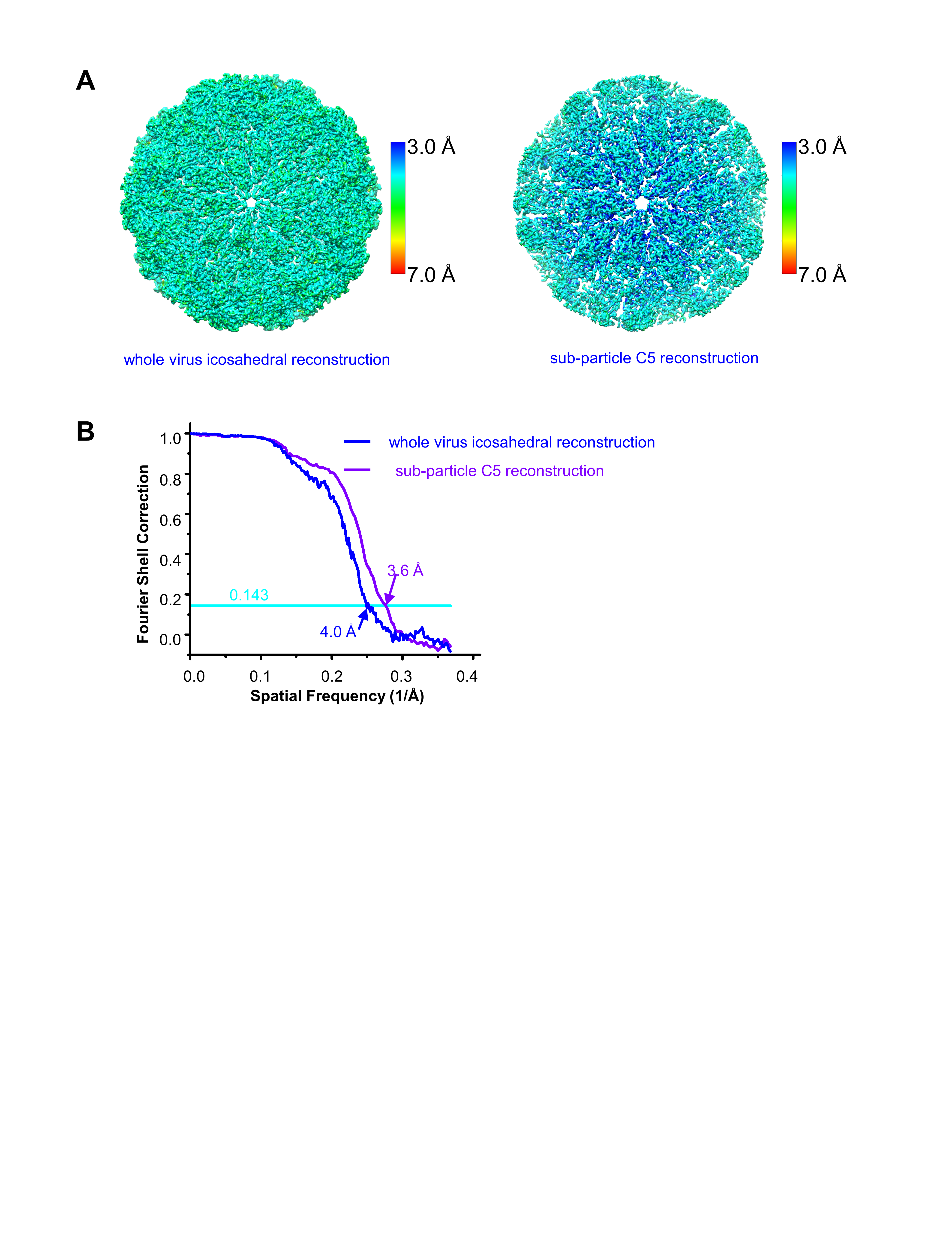

Supplement: FIG S1 [file mBio.02924-20-sf001.tif]

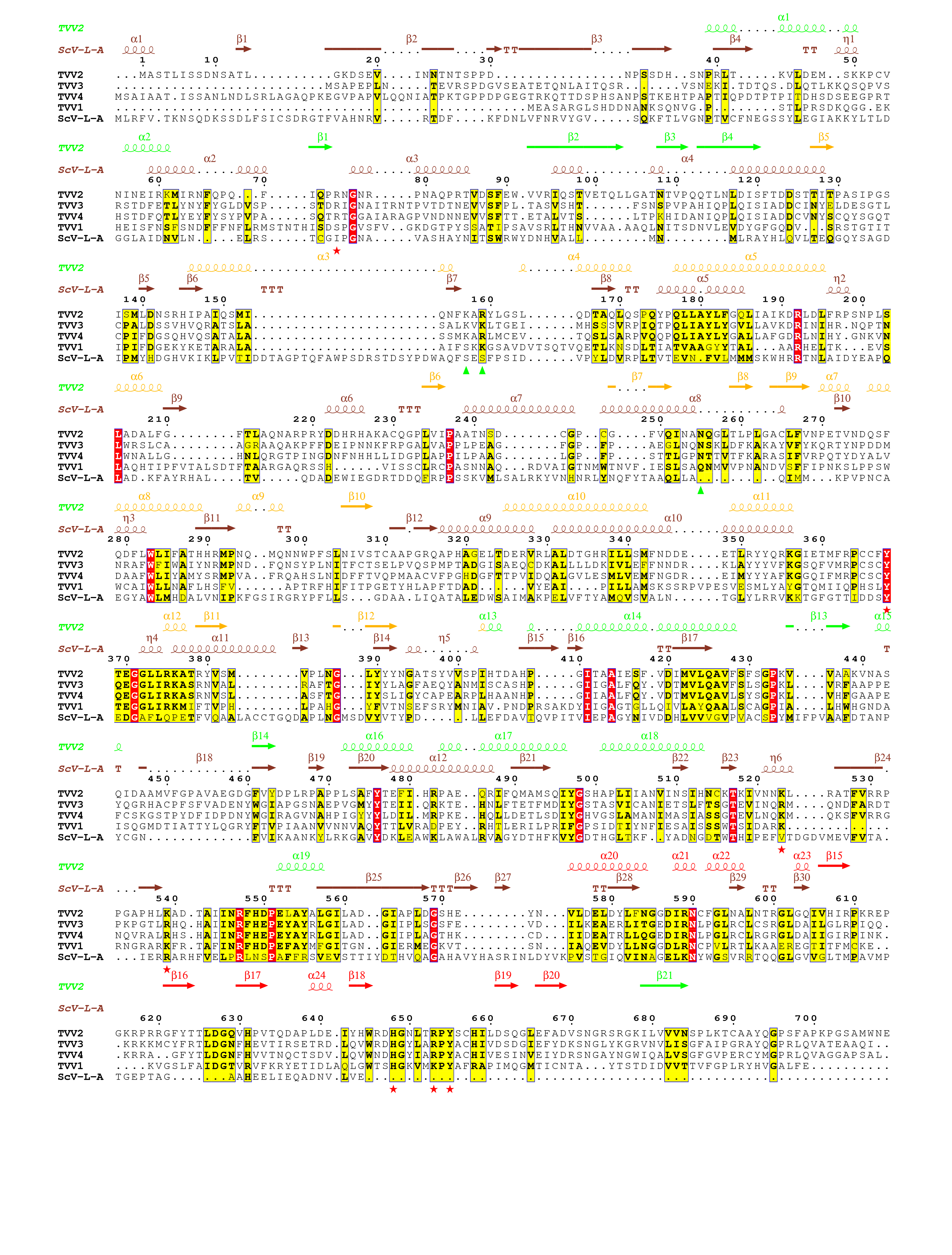

Supplement: FIG S2 [file mBio.02924-20-sf002.tif]
